# Insight into miRNA biogenesis with RNA sequencing

**DOI:** 10.18632/oncotarget.5264

**Published:** 2015-08-26

**Authors:** Thomas Conrad, Ulf Andersson Ørom

**Affiliations:** Max Planck Institute for Molecular Genetics, Berlin, Germany

**Keywords:** microRNA, microprocessor

MicroRNAs (miRNA) are small RNAs that posttranscriptionally regulate gene expression, predominantly by modulating the translation and stability of target messenger RNAs [1]. Over the last 15 years, a tremendous expansion of knowledge has changed the perception of miRNAs from a rare peculiarity in nematode worms to a ubiquitous layer of gene expression regulation, with broad roles in development, and cellular and organismic homeostasis across animal and plant species. Accordingly, aberrant miRNA expression is a hallmark of various severe disease phenotypes such as cardiomyopathies or cancer.

Nevertheless, despite extensive efforts to dissect miRNA functions, many open questions are still remaining. Especially the upstream events that regulate processing have recently received increasing attention, as the DNA elements that control miRNA expression and the cellular signaling pathways that fine-tune miRNA processing are still poorly characterized.

One aspect of the miRNA biogenesis pathway that remains challenging is to understand why these short RNAs are expressed as long primary transcripts (pri-miRNA) that can be several kilobases in length. While several attempts have been made to predict the transcription start sites (TSS) of pri-miRNAs, their validation remains an experimental challenge due to their nuclear localization and decreased stability compared to mRNAs.

pri-miRNAs are processed co-transcriptionally to precursor miRNA hairpins (pre-miRNA) by the Microprocessor complex. pre-miRNAs are then further processed by Dicer into mature miRNAs and incorporated into the RNA-induced silencing complex (RISC) to exert their functions [2]. At the same time, the complex can distinguish pri-miRNA transcripts from unrelated RNA stem-loop structures through unknown mechanisms, likely dependent on sequence elements in the flanking regions.

In our recent work we show that the endogenous Microprocessor activity towards individual pri-miRNAs can be determined using RNA sequencing of chromatin-associated RNA and random primed library generation [3]. Using this approach we identify the Microprocessor cleavage signature, a pronounced dip in the sequencing reads of chromatin bound pri-miRNAs reminiscent of the cleaved-out pre-miRNA hairpin. Based on the extent of this signature, we define the MicroProcessing Index (MPI) as a measure for processing efficiency. Genome-wide assessment of the MPIs of all expressed pri-miRNAs shows that processing is one of the major determinants for the expression level of individual mature miRNAs, exceeding the contribution from transcriptional or post-processing regulation. The processing efficiency of specific pri-miRNAs is comparable between cell lines, suggesting a major impact of primary sequence and RNA structure, and to a lesser extent a differential regulation by cell-type specific co-factors. We can derive sequence motifs associated with an increase in processing efficiency, partially confirming previous *in vitro* approaches [4], particularly the CNNC motif (where N is any base) 3′ of the pre-miRNA that has been shown to be important for processing of *C*. *elegans* pri-miRNAs in human (4). This *in vitro* study suggested that proteins such as the SR protein SRP20 can bind to the regions flanking the pre-miRNAs and facilitate processing [4].

The aspect of pri-miRNA TSS was not further addressed in our paper, but it is evident from the data that the accurate definition of the entire pri-miRNA transcript is feasible for abundant pri-miRNAs, as has also been reported by a recent paper applying a similar approach but with oligod(T) priming in the RNA sequencing library preparation [5].

Large-scale sequencing studies keep providing new insights into the general mechanisms of pri-miRNA transcription and processing as it occurs in a cellular context. Two papers published this year use NET-seq to study global transcription by RNA polymerase(II) [6, 7]. These data have also been suggested to provide insight into pri-miRNA processing events that occur co-transcriptionally. From the data it is evident that transcriptional pausing or intermediate cleavage events occur at pre-miRNA sites at chromatin. While the interpretation of these data is not yet conclusive with respect to miRNA expression, they could reveal possible links between pri-miRNA processing and the transcription process.

In summary, large-scale approaches using RNA sequencing are proving to be valuable to uncover some of the missing aspects of pri-miRNA transcription and processing, and more assays like these are expected to add further to our understanding of miRNA biogenesis in the near future.
